# Potential Role of Xylitol Plus Grapefruit Seed Extract Nasal Spray Solution in COVID-19: Case Series

**DOI:** 10.7759/cureus.11315

**Published:** 2020-11-03

**Authors:** Camille Celeste Go, Krunal Pandav, Marcos A Sanchez-Gonzalez, Gustavo Ferrer

**Affiliations:** 1 Family Medicine, Larkin Hospital Palm Springs Campus, Hialeah, USA; 2 Division of Clinical Research and Academic Affairs, Larkin Community Hospital, South Miami, USA; 3 Division of Research and Academic Affairs, Larkin Community Hospital, South Miami, USA; 4 Pulmonary and Critical Care, Aventura Hospital and Medical Center, Aventura, USA

**Keywords:** grapefruit seed extract, xylitol, covid-19, sars-cov-2, intranasal, therapeutics

## Abstract

The SARS-CoV-2 virus has created an unprecedented impact on healthcare globally. Being a novel virus, several treatments have been explored against COVID-19. During the early stages of the disease, treatment is mainly supportive. While several studies have suggested different treatment modalities, there is still no definitive treatment against COVID-19. Re-purposing already established medications, with excellent safety profiles, is a possible approach for treating the disease in its early stage. Having a mode of transmission as a droplet mode, several studies have supported how the nose can contain the primary route of entry of SARS-CoV-2. Hence, we postulated that re-purposing a commercially available nasal spray containing xylitol and grapefruit seed extract (GSE), namely Xlear Nasal Spray® (Xlear, Inc., American Fork, USA) could be used as an adjunct treatment of COVID-19. With a well-established safety profile, the components of this nasal spray have been studied and have been shown to have potential efficacy against viral pathogens, including coronavirus, and may potentially regulate pathways important in the initial entry of infection, replication, and systemic response to SARS-CoV-2. We present a series of three mild-moderate risks, symptomatic, COVID-19 patients, treated with the intranasal combination, as an adjuvant to their ongoing treatment, with rapid clinical improvement and shorten time to negativization on repeat intranasal swab test via PCR. No safety issues were noted during the course of treatment. Xlear nasal spray, containing xylitol plus GSE, given its established safety profile and compelling clinical results described here, could be a potential adjunct treatment option in mild-moderate COVID-19 cases.

## Introduction

Severe acute respiratory syndrome coronavirus 2 (SARS-CoV-2) is a novel virus and the causative agent of the Coronavirus disease 2019 (COVID-19). Currently, the management and therapeutic options for COVID-19 are limited, including self-quarantine and supportive care, usually indicated for mild cases. In contrast, a moderate disease in high-risk patients and patients with severe conditions generally require hospitalization [1]. COVID-19 has caused a significant impact on the healthcare systems of various countries across the globe. As of October 3, 2020, COVID-19 has infected over 34,790,000 and caused over 1,031,000 deaths worldwide. In the United States alone, over 7,379,000 COVID-19 cases and over 200,000 deaths have been reported [2]. This situation has led to an urgent need for therapeutic options prompting increased interest in re-purposing the existing medications that might play a role in the treatment of COVID-19. Interestingly, it has been documented that both angiotensin-converting enzyme 2 (ACE2) and transmembrane serine protease 2 (TMPRSS2), which are present not only in the bronchial epithelium and alveolar type II epithelium cells but also in the nasal epithelium, are associated with the virus entry into the cell. Pharmacological agents such as nasal sprays might be optimal therapeutic candidates for providing better outcomes in COVID-19 patients if used in the early stages of the disease [1-3]. In their systematic review, Gengler et al. have reported that viral shedding appears to be largely from the nasal cavity further suggesting that the nasal cavity could be a major source of COVID-19 transmission. Moreover, the nasal shedding could place various healthcare workers, such as the ones participating in rhinologic procedures, at a higher risk of COVID-19 [4].

Prior studies suggest that candidate agents with potential activity against SARS-CoV-2, which can be administered intranasally, might play a pivotal role in the treatment against COVID-19. In this vein, we have identified two candidate agents with potential activity against SARS-CoV-2 which can be administered intranasally, namely, xylitol and grapefruit seed extract (GSE). Moreover, the antiviral effects of xylitol as well as its antimicrobial properties are evaluated and have been documented [5]. Properties of GSE were evaluated in vitro with several viruses such as the avian influenza virus (AIV), Newcastle disease virus (NDV), infectious bursal disease virus (IBDV) and it showed activity against enveloped viruses such as the AIV and NVD but resistance with non-enveloped viruses namely IBDV [6]. In a study conducted by Bansal et al., they concluded that out of three samples of iota-carrageenan, all three were effectively able to inhibit the SARS-CoV-2 [5]. The third sample containing xylitol in combination with addition to iota-carrageenan was able to demonstrate an antiviral effect against SARS-CoV-2 at various concentrations which were tested in the study suggesting its potential role in COVID-19 [5]. Preliminary studies conducted by our group at two different laboratories have tested the individual components xylitol and GSE of commercially available intranasal spray (Xlear Nasal Spray®; Xlear, Inc., American Fork, USA). The results of the aforementioned preliminary studies seem to point toward the conclusion that the components exert a significant virucidal effect.

Accordingly, taking into consideration the current COVID-19 pandemic situation and the antimicrobial and virucidal effects of xylitol and GSE, we hereby present a case series of three COVID-19 positive patients who were prescribed the commercially available Xlear nasal spray, which contains xylitol and GSE, for a duration of seven days. The present cases highlight the potential efficacy of intranasal xylitol and GSE, as a therapeutic aid for the management and treatment of COVID-19.

## Case presentation

Case 1

A 16-year-old Hispanic female, with a past medical history of iron deficiency anemia, hemoglobin levels unknown, and was treated previously with ferrous sulfate, which was diagnosed three years ago, tested positive for COVID-19 on July 7, 2020. The patient is a non-smoker, with no past surgical history and not taking any maintenance medications. The patient complained of sore throat, dry mouth, nasal congestion, runny nose, productive cough with yellow sputum, anosmia, and ageusia, in addition to reporting waking up at night due to the coughing episodes, two days before consultation. Afebrile, no abdominal pain, diarrhea, no shortness of breath, weakness, or lethargy were reported. The patient also reported taking self-medication for two days with warm water and tea, which did not help alleviate the symptoms. A consultation with a primary care physician was pursued followed by a COVID-19 reverse transcriptase-polymerase chain reaction (RT-PCR) test via nasopharyngeal swab performed on the patient. Two days later, the patient tested positive, subsequently enrolled in this case series, and was given the experimental treatment. The patient was instructed to spray Xlear nasal spray twice per nostril four times a day every six hours for seven days, which was an adjunct to her self-medication. The patient continued to self-medicate with warm water and tea, and supportive treatment. On day 1, the patient complained of a stuffy nose, anosmia, ageusia, tiredness, cough, stuffiness, and congestion. Oxygenation 98% on room air, pulse rate 78 beats per minute, afebrile, with mild symptoms on Symptoms Assessment Score (SAS). Patients rated generalized pain as three on the Visual Analogue Score (VAS) and the Numerical Rating Scale (NRS). On day 3, an improvement was noted in her symptoms, particularly anosmia which she reported being able to smell strong substances. On average, the documented resolution of anosmia is two weeks [7]. An improvement of cough was also noted. The patient’s labs were drawn on day 4 with normal levels of c-reactive protein (CRP) and d-dimer (Appendices). On day 7, the patient noted an improvement in the overall symptoms with a reduced degree of tiredness, absence of cough, congestion, and stuffiness. Although mild ageusia is still present, it was markedly improved compared to day 1. The patient remained afebrile throughout the duration of the trial. Improvement of symptoms was noted during the trial duration. On day 7, the patient was retested for COVID-19 RT-PCR via nasopharyngeal swab with non-reactive results. Repeat testing of COVID-19 RT-PCR was also done on day 8 which also showed non-reactive results (Appendices). A follow-up was done on day 14 and the patient reported no symptoms with a return to baseline health.

Case 2

A 60-year-old Hispanic male was tested positive for COVID-19 on July 7, 2020. The patient had a past medical history of leukemia, which was diagnosed in 2012. Currently in remission, post-chemotherapy and radiation therapy, a heavy smoker, and occasional alcoholic beverage user. The patient reported using maintenance medications: zolpidem 10 mg once a day and bupropion HCL 100 mg two times a day. Two days before consulting the primary care physician, the patient started to experience sore throat, dry mouth, sneezing, nasal congestion, runny nose associated with anosmia and ageusia, and a low-grade fever at 101 Fahrenheit (F). No abdominal pain, diarrhea, shortness of breath weakness, or increased tiredness were noted. Furthermore, self-medication with warm water and tea did not help alleviate the symptoms that were reported by the patient. Upon consultation with the primary care physician, the patient was tested for COVID-19 RT-PCR via the nasopharyngeal swab. Following the positive COVID-19 test, the patient was subsequently enrolled in the case series experimental group. The patient was instructed to use the Xlear nasal spray four times a day every six hours for seven days, which was an adjuvant to his self-medication. The patient self-medicated with acetaminophen for fever, multivitamins, tea, and warm water. On day 1, the patient complained of a stuffy nose, sneezing, congestion, sandy and watery eyes, with oxygen saturation at 97% room air and pulse rate of 86 beats per minute. The patient also complained of anosmia, fever, 101 F. Rated overall symptoms as mild on SAS and generalized pain as three VAS and NRS. On day 2, the patient was noted to have tiredness and productive cough, with awakenings at night time due to coughing episodes. The patient also noted increasing tiredness. The patient also complained of ageusia. However, the patient was now afebrile with stable oxygenation and pulse rate. On day 3, the patient noted improvement of symptoms with only sandy eyes, anosmia, and ageusia. The patient also noted that he was now able to start smelling strong substances. On day 4, the patient's CRP and d-dimer were tested and were unremarkable (Appendices). On day 7, the patient reported symptoms of tiredness, ageusia, and anosmia with a 70-80% improvement of the ability to smell. The patient remained afebrile starting on day 2 with noted improvement during the entire seven days. Subsequently, on the same day, the patient was retested for COVID-19 with RT-PCR via nasopharyngeal swab with non-reactive results. The patient continued to smoke 10 tobacco sticks per day throughout the duration of the trial. A repeat test for COVID-19 RT-PCR was done and yielded a negative result (Appendices). A follow-up was done on day 14 and the patient reported no symptoms with the return to baseline health.

Case 3

A 38-year-old Hispanic male tested positive for COVID-19 on September 26, 2020. The patient had a past surgical history of arthroscopy 20 years ago and was currently taking n-acetyl glucosamine, vitamin C, and vitamin D daily. The patient has grade 1 obesity (body mass index 30), a non-smoker, non-alcoholic beverage drinker, and does not use illicit drugs. Two days before the consultation in urgent care, the patient started to experience cold-like symptoms, sinus pressure, night sweats, and unquantified fever. Consultation at urgent care was then pursued. On the chest X-ray, diminished lung volumes with hypoventilatory changes at the lung bases with basilar atelectasis were noted. The patient was prescribed azithromycin 250 mg for five days along with albuterol nebulization (Appendices). Five days after the consultation, the patient continued to feel worse despite taking the prescribed medications. The patient then opted to have himself tested for COVID-19 after learning that his uncle, whom he interacted with a few days prior, tested positive for COVID-19. The patient subsequently tested positive. The patient was then prescribed with Xlear nasal spray and was instructed to spray twice per nostril four times a day every six hours for seven days, as an adjuvant to his ongoing previously mentioned treatment. On day 1, the patient complained of a runny and stuffy nose, tiredness, productive cough, nasal congestion, diarrhea, with oxygen saturation at 94%, and afebrile. The patient also complained of headache, rated two on the VAS and NRS scale, and rated overall symptoms as mild. On day 3, the patient noticed an improvement of symptoms with only tiredness, nasal congestion, and cough. The patient demonstrated stable vital signs and oxygenation and reported that he has regained his sense of smell and his sense of taste. On day 4, the patient's CRP and d-dimer were tested and were unremarkable (Appendices). On day 7, the patient reported symptoms of tiredness, and cough, although overall symptoms significantly improved as compared to previous days. The patient remained afebrile throughout the trial with noted improvement in all seven days. The patient was also retested for COVID-19 with RT-PCR via nasopharyngeal swab and showed non-reactive result (Appendices). Follow-up was done on day 14 and the patient reported no symptoms with a return to baseline health. No repeat chest X-ray was done post-treatment.

## Discussion

These reported cases are the first to shed some light regarding the potential efficacy of utilizing intranasal xylitol plus GSE as an adjunct treatment against COVID-19 and reduction to the time of negativization on nasal RT-PCR. While it is difficult to have definitive proof of efficacy in a form of a case series, we believe that the present series provides a rationale for initiating larger randomized placebo-controlled clinical trials evaluating the utilization of xylitol plus GSE in the form of an intranasal spray in COVID-19 patients.

The patients we reported also had risk factors that could increase the risk of morbidity and mortality: patient 1 had iron deficiency anemia, corrected with iron therapy; patient 2 had a significant risk factor given his smoking status and history of cancer, chemotherapy, and radiation; and patient 3, although relatively healthy, was mildly obese with history of arthroscopy. The above-mentioned patients have a higher than average risk of COVID-19 morbidity and mortality [8]. Neither of the patients progressed to severe disease and all patients showed improvement in the symptoms with the intranasal use of xylitol plus GSE, with a reduced number of days to testing positive to negative via COVID-19 RT-PCR nasal swab test.

Two sprays per nostril every six hours were administered to these three patients. A standard dose per nasal spray contains up to 140 µL per spray. Based on the nasal cycle, each dose of 140 µL per spray delivered properly into the nasal cavity with an estimated nasal airway surface liquid volume in the range of 50-375 µL should remain in the cavity an average of four to six hours [9-14]. A study conducted by Hou et al. stated that there is a strong association between the high levels of ACE2 and SARS-CoV-2 infectivity [15]. Studies show a higher ACE2 level in the nasopharyngeal tract compared to the lower respiratory tract [15,16]. In a genomic map of COVID-19 by the University of North Carolina at Chapel Hill, it was found that there is a gradient with greater expression of ACE2 receptors and SARS-CoV-2 infectivity in the nose compared to the peripheral lung tissue. These case series support our rationale that therapeutic strategies should aim at reducing viral load in the nose early in the disease using nasal sprays or lavages [17]. The underlying role of xylitol and GSE on influencing the ACE2 levels in the nasopharyngeal tract remains to be elucidated.

Lastly, the time to negativization is important to note. The average time to negativization was found to be approximately an average of 14 days [18]. These cases have shown that by using xylitol plus GSE in the form of an intranasal spray (Xlear nasal spray), as an adjunct to the ongoing treatment, the time to negativization was reduced to seven days, a 50% reduction compared to the usually known average. It should be noted that in this case series, a combination of intranasal xylitol plus GSE nasal spray was mainly used as an adjunct therapy to ongoing treatments for COVID-19.

## Conclusions

In summary, the three patients reported in this article, with minimal to moderate risk for morbidity and mortality from COVID-19, demonstrated an improvement in the symptoms and a reduction in the clinical course post use of xylitol plus GSE in the form of a nasal spray, commercially available as Xlear nasal spray, as an adjunct to their ongoing treatment. This combination could play a potential role in improving the outcome in mild to moderate COVID-19 patients. While relatively safe for general use, larger randomized, placebo-controlled clinical trial studies are mandated which could shed further light on this topic.

## Figures and Tables

**Figure 1:**
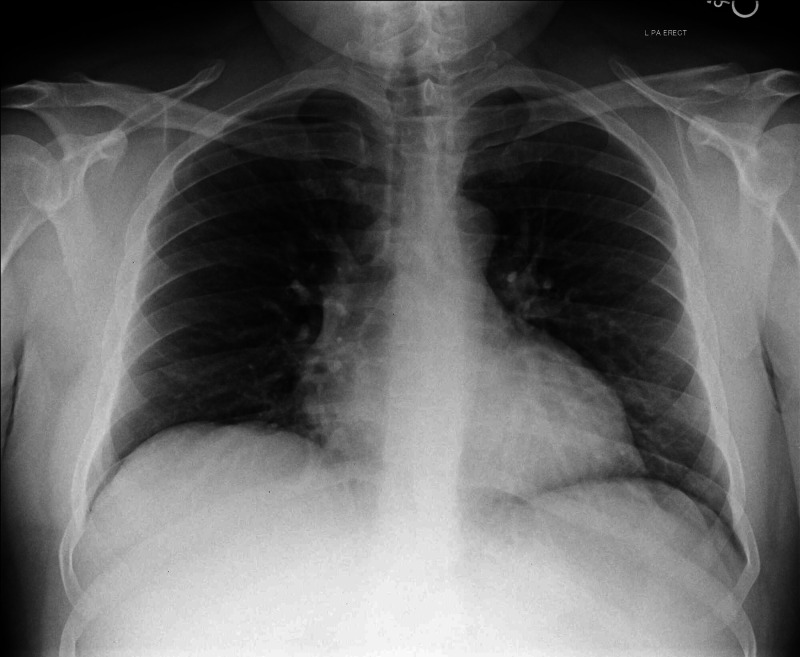
Chest X-ray image of patient 3

## Appendices

**Table 1:** Laboratory results of patients. CRP: C-reactive protein, RT-PCR: reverse transcriptase-polymerase chain reaction.

Day	Laboratory Test	Normal Values	Patient 1	Patient 2	Patient 3
Day 4	CRP	0.0-9.9 mg/L	0.4	0.4	0.4
Day 4	D-Dimer	<0.50 mg/L	0.19	0.19	0.19
Day 7	COVID-19 RT-PCR	Non-reactive	Non-reactive	Non-reactive	Non-reactive
